# Effect of synbiotics on growth performance, gut health, and immunity status in pre-ruminant buffalo calves

**DOI:** 10.1038/s41598-023-37002-6

**Published:** 2023-06-22

**Authors:** Amit N. Sharma, Parul Chaudhary, Sachin Kumar, Chand Ram Grover, Goutam Mondal

**Affiliations:** 1 Animal Nutrition Division, National Dairy Research Institute, Karnal, Haryana 132001 India; 2 School of Agriculture, Graphic Era Hill University, Dehradun, Uttarakhand 248002 India; 3 Dairy Microbiology Division, National Dairy Research Institute, Karnal, Haryana 132001 India

**Keywords:** Microbiology, Diseases, Gastroenterology

## Abstract

Synbiotics are employed as feed additives in animal production as an alternate to antibiotics for sustaining the gut microbiota and providing protection against infections. Dairy calves require a healthy diet and management to ensure a better future for the herd of dairy animals. Therefore, the present study was carried out to investigate the effect of synbiotics formulation on growth performance, nutrient digestibility, fecal bacterial count, metabolites, immunoglobulins, blood parameters, antioxidant enzymes and immune response of pre-ruminant Murrah buffalo calves. Twenty-four apparently healthy calves (5 days old) were allotted into four groups of six calves each. Group I (control) calves were fed a basal diet of milk, calf starter and berseem with no supplements. Group II (SYN1) calves were fed with 3 g fructooligosaccharide (FOS) + *Lactobacillus plantarum* CRD-7 (150 ml). Group III (SYN2) calves were fed with 6 g FOS + *L. plantarum* CRD-7 (100 ml), whereas calves in group IV (SYN3) received 9 g FOS + *L. plantarum* CRD-7 (50 ml). The results showed that SYN2 had the highest (*P* < 0.05) crude protein digestibility and average daily gain compared to the control. Fecal counts of *Lactobacilli* and *Bifidobacterium* were also increased (*P* < 0.05) in supplemented groups as compared to control. Fecal ammonia, diarrhea incidence and fecal scores were reduced in treated groups while lactate, volatile fatty acids and antioxidant enzymes were improved compared to the control. Synbiotic supplementation also improved both cell-mediated and humoral immune responses in buffalo calves. These findings indicated that synbiotics formulation of 6 g FOS + *L. plantarum* CRD-7 in dairy calves improved digestibility, antioxidant enzymes, and immune status, as well as modulated the fecal microbiota and decreased diarrhea incidence. Therefore, synbiotics formulation can be recommended for commercial use in order to achieve sustainable animal production.

## Introduction

Dairy calves require a balanced diet and proper management to ensure a better future for animal health and production. The gastrointestinal tract (GIT) of calves is sterile prior to parturition, but the neonate’s tract is colonized by microbes from the surrounding environment and birth canal^1^. The GIT microbiota of newborn calves is highly sensitive to changes in diet, environment, disease, and stress that affect the microbial flora. The GIT serves as the main organ for nutrient absorption and acts as the first line of defense until the immune system cells have fully developed^2^. It is estimated that about 20% calf mortality rate in herds can reduce net earnings by up to 40%. Neonatal diarrhea is the primary cause of death in calves throughout their pre-ruminant lives in the dairy sector^3^. Antibiotics have long been used to prevent and treat gastrointestinal infections in dairy animals. However, indiscriminate use of antibiotics has resulted in the development of antibiotic-resistant bacteria, which has long-term consequences, as well as the destruction of healthy gut microflora^4^. As a result, AMR-free feeding is required for safe and healthy livestock production.

Prebiotics and probiotics and their combinations could be used as a substitute to treat GI illnesses and boost the host’s immune function. Prebiotics are oligomers that cannot be metabolized by digestive enzymes and can thus be used by gut microbes to accelerate their growth and development^5^. They promote the growth and activity of beneficial bacteria by protecting the intestinal walls from pathogens and reducing microbe expansion in the GI tract^6^. Carbohydrate substrates such as oligosaccharides or dietary fibers are the most commonly used prebiotics for health benefits. Fructo-oligosaccharides (FOS) and spray-dried bovine serum were used to minimize the incidence of GI illness in calves by preventing the adhesion of *Escherichia coli* and *Salmonella*^7^. Cellooligosaccharide supplementation improved calve’s feed efficiency by improving ruminal fermentation as propionate and total fatty acids levels in the postweaning period^8^. Oligosaccharides may be helpful in controlling rumen fermentation by increasing protein and VFA levels while decreasing the ammonia nitrogen^9^. Applying galactic-oligosaccharides improved feed efficiency, serum high-density lipoprotein, and decreased diarrhea incidence in dairy calves^10^. According to a meta-analysis, dairy calves supplemented with mannan-oligosaccharide (Bio-Mos®) improved body weight gain^11^.

Probiotics are live beneficial microorganisms that protect GI from pathogens and stimulate bactericide production against pathogens^12^. Probiotics have recently been suggested to improve animal health, growth performance, nutrient digestibility, gut microbial balance, and immune responses^13^. Besides, probiotics have been shown to improve the equilibration of an animal’s beneficial microbial population by boosting the host immune response via appropriate secretions of IgA, interleukins, and competitive exclusion of potentially harmful bacteria in the digestive system^14^. *Lactobacillus* probiotic stimulated the production of pro and anti-inflammatory cytokines, regulated immune response, and improved growth performance, nutrient digestibility, and reduced stress in calves^15^. Probiotic supplementation reduced *Escherichia* while increasing the natural microbial flora, including lactic acid bacteria and the *Bifidobacterium* population^16^. Multispecies probiotic mixture in feed improved host immunity and growth performance in dairy calves^17^. Wang et al.^18^ reported that probiotic consortia (*L. plantarum, Pediococcus acidilactici, P. pentosaceus* and* Bacillus subtilis*) added to the calf’s diet affected rumen fermentation, improved calve’s immunity as well as health status, and decreased fecal score at 3 weeks of age. Lactic acid bacteria supplementation also improved weight gain, feed efficiency, and calve’s health^19^. *Bacillus amyloliquefaciens* decrease volatile fatty acid synthesis and inhibits taste in pellet feed, which allows ruminants to consume more feed^20^. *Saccharomyces cerevisiae* was supplemented to calf GIT after birth, which raised the abundance of beneficial bacteria in the intestinal microbiota and improved immunity and intestinal homeostasis^21^.

Synbiotics are a prebiotics and probiotics combination that may synergistically benefit host health than either of the probiotics or prebiotics alone by improving the survival and colonization of beneficial microorganisms in GIT. The combination of prebiotic inulin and *Enterococcus faecium* can help postnatal rumen development and improve its functionality^22^. Kormomix® Rumin, a commercial synbiotic added to nursing cow’s feed, improves rumen fermentation, and allows animals to consume their feed more efficiently without having an impact on their blood parameters^23^. Inulin and *S. cerevisiae* supplementation improved the rumen development and digestive canal of Holstein crossbred calves^24^. However, there is a scarcity of research on the effect of synbiotics supplementation on pre-ruminant calves and their potential to compete with harmful microbes. Therefore, the aim of the present study was to investigate the effect of synbiotics formulations on dry matter intake, growth performance, blood parameters, antioxidant enzymes, beneficial and harmful bacterial population, and diarrheal incidence in Murrah buffalo calves.

## Material and methods

### Ethical approval

The present study was conducted in the Livestock Research Centre, National Dairy Research Institute, Karnal, Haryana, India, and accomplished under the fundamental guidelines for the proper conduct of animal experiments and related activities as per the standard of the Institute Animal Ethics Committee (IAEC). This study was approved by IAEC with approval no. 41-IAEC-18-18. This study was conducted for a total period of 75 days. The study is reported in accordance with ethical arrival guidelines.

### Synbiotics preparation

The pure culture of *L. plantarum* CRD-7 strain (NCBI accession no. KJ769142) was procured from Synbiotic Functional Foods Laboratory, Dairy Microbiology Division, NDRI, Karnal. The obtained culture was activated in MRS broth, which contained 10^8^ cfu/ml. Murrah buffalo milk was used to make fermented milk which was heated for 10 min at 85 °C before being chilled at room temperature. The mixture was incubated for 7–8 h at 37 °C with *L. plantarum* CRD-7 (1%). The FOS was added to it just before the feeding. The FOS utilized in this study had a bulk density of 700 g/L, purity of 97.50%, and conductivity of 28 g/100 g (µS/cm), and it was acquired from Beneo, Sudzucker group, Belgium.

### Experimental design

Twenty-four pre-ruminant Murrah buffalo calves (5 days old) were randomly divided into four groups with six calves in each group using randomized block design (RBD). Group I (control, CON) calves were fed a basic diet consisting of milk, calf starter, and berseem with no supplements. Group II (SYN1) calves were fed with 3 g FOS in fermented milk (150 ml) containing *Lactobacillus plantarum* CRD-7. Group III (SYN2) were fed with 6 g FOS in fermented milk (100 ml) containing *L. plantarum* CRD-7, while group IV (SYN3) calves were fed with 9 g FOS + *L. plantarum* CRD-7 in the form of fermented milk (50 ml) having 10^8^ cfu/ml/calf/day with basal diet.

Individual calf pens were well-ventilated, with appropriate heating to protect them from the cold temperature. The calf cages were kept well maintained and in hygienic condition by cleaning the adjoining area twice a day. The calf was kept at 18–21 °C, air changes (8–12 per hour), humidity (35–70 °C), and lightning (12 h light/dark cycle) to provide a comfortable environment for the animals.

### Management of calves

Milk was provided to the animals twice a day. Whole milk was given to the calves at a rate of 1/10 of BW for the first 2 weeks, 1/15th of BW for the third and fourth weeks, and 1/20th BW for the final 8 weeks of the experimental trial. Beginning with the first week, calf starters were made available. All of the calves were fed with concentrate and green ad libitum. The calf was offered berseem as green fodder. All the calves had unlimited access to fresh water at all times. All the groups were fed as per ICAR guidelines^25^.

### Analysis of feed and dry matter intake

The experimental period lasted a total of 75 days. The calf’s feed intake was monitored daily and was calculated and expressed as average values. After 56 days of experimental feeding, a 5-day digestibility trial was conducted to assess nutrient digestibility. The dry matter intake (DM), organic matter (OM), ether extract (EE), crude protein (CP), and neutral and acid detergent fiber (NDF and ADF) were determined according to Refs.^26,27^.

### Body weight changes and morphometry parameters

Calve’s body weight was recorded as an initial body weight on the first day of the experiment and subsequently monitored at weekly intervals till the end of the experimental trial. The difference between the starting and final body weights was used to compute total weight gain. Body and hip height, heart girth, and length were measured with measuring tape to keep track of skeletal measurements. Dry matter intake was recorded and estimated by different groups every day to calculate the difference between provided and leftovers by the calves. A 5-day digestibility trial was conducted to assess nutrient utilization.

### Blood parameters

Blood samples were collected from buffalo calves at the beginning (0 days), middle (30 days), and after 60 days of the experiment in the early morning before feed. The samples were taken from animals into a vacutainer containing heparin. Instantly, the vials were slightly rolled among the palms for proper mixing, kept in an ice box, and carried into a laboratory for further analysis. Blood haematology parameters such as total leucocyte count (TLC), hemoglobin (Hb), packed cell volume (PCV), total eosinophil count (TEC), and differential leukocyte count (DLC) using a Vet blood analyzer. The serum glucose, total protein, albumin and globulin were estimated using spectrophotometric methods (Spectro UV–VIS dual beam, CA, USA). An Enzyme-Linked Immunosorbent assay kit (Bioassay Technology Laboratory Cat no. E0009B0 and E0010B0) was used to measure IgA and IgG levels^28–30^.

### Estimation of antioxidant enzymes

The RBC hemolysate prepared from freshly drained blood was tested for antioxidant activity. Madesh and Balasubramanian^31^ described the method for estimating superoxide dismutase (SOD), which involved the production of superoxide via pyrogallol autooxidation and reduction of the tetrazolium dye to formazan at 570 nm. Catalase activity was determined using the method described by Aebi^32^.

### Estimation of humoral and cell-mediated immunity

The humoral immune response was used to determine antibody titer against chicken RBC (CRBC), the animal was treated for 30 days with a single dosage of chicken erythrocyte in phosphate buffer saline (PBS, 10%) administered intravenously in animals (1 ml). Samples of blood were collected before the inoculation (0d) and then at 7, 14 and 21 days after the challenge to estimate the antibody response^33^. Approximately 2 ml of blood was collected in sterile serum collection vacationers, and collected serum was transferred aseptically to washed marked plastic vials and stored at − 20 °C until further analysis by the hemagglutination (HA) test. All the calves were injected with phytohemagglutinin-P (150 µg PHA-P) intra-dermally in the neck to measure the cell-mediated immune response in terms of delayed-type hypersensitivity (DTH)^34^. The DTH response was measured using Verniers calliper and expressed as the percent increase in skin thickness.

### Faecal collection and sampling

Faecal samples were collected from the rectum using manual stimulation at 0, 30, and 60 days. To avoid injury, sterile latex gloves and glycerine were used to collect 10–12 g of faeces to analyse pH, ammonia, lactic acid, VFA, and microbial population, respectively. A sterile container held the sample and stored it at −4 °C for further analysis. Before aliquoting the faeces, the pH of the faeces was evaluated using a digital pH metre (Eutech, Klang Selangor, Malaysia).

For the determination of fermentative products (lactic acid, ammonia and VFA), three aliquots were prepared. Approximately 2 g of fresh faeces was acidified with 6 N HCL (6 ml) and stored at 20 °C. This supernatant (5 ml) and NaOH (10 ml) were steam distilled by KELPLUS-N analyser (Pelican, India). Ammonia was gathered in a solution of boric acid (20%) and mixed indicator and titrated against known strength of H_2_SO_4_.

For lactic acid analysis, an aliquot (2 g) was mixed with distilled water (4 ml), centrifuged at 10,000 rpm, and the supernatant was kept at 20 °C. A third aliquot of 2 g of fresh faeces was mixed with metaphosphoric acid (25%), centrifuged for 10 min at 10,000 rpm, and the supernatant was kept at 20 °C for VFA analysis. The individual VFA in various samples was calculated by a gas chromatograph (Nucon 5700, India) outfitted with a flame ionisation detector. The injector port, column and detector temperature (210, 180 and 230 °C) were the analytical conditions for fractionating VFA. Carrier nitrogen gas flowed at a rate of 40 ml/min^35^.

### Microbial count from faecal using different media

One gram of faecal sample was mixed with 9 ml normal saline solution, then serially diluted from 10^−1^ to 10^−8^. One ml diluted sample was pour plated into MRS media, *Bifidobacter* agar, Eosin methylene blue agar and Clostridium agar medium. Plates were incubated at 37 °C for 24 h, while plates were incubated in an anaerobic jar for *Bifidobacterium* and *Clostridium* count. Colony-forming units were counted using a colony counter and expressed as log 10 cfu/g^36^.

### Diarrheal incidence and faecal score

The faecal consistency score in calves was noted every single day during the experiment. A number of diarrhoea sieges (2 or more consecutive days with scores of 4 s or three or more consecutive days with scores of 3 s, or 3 s and 4 s)^36^.

### Statistical analysis

Results were statistically analysed using SPSS software ver. 16.0 through analysis of variance at *P* < 0.05. Some of the parameters were analysed (nutrient intake and digestibility) using one-way analysis of variance. Morphometry parameters were analysed using repeated measure analysis and two-way ANOVA. Data for blood parameters, antioxidants enzyme, microbial count and faecal metabolites after different time interval were analysed using two-way ANOVA by descriptive statistics at *P* < 0.05. The parameters values were shown as mean ± standard error. Time/period and treatments were fixed effects, with no random effects because the animals were similar in nature.

## Results

### Nutrient intake and digestibility

**Figure 1:**
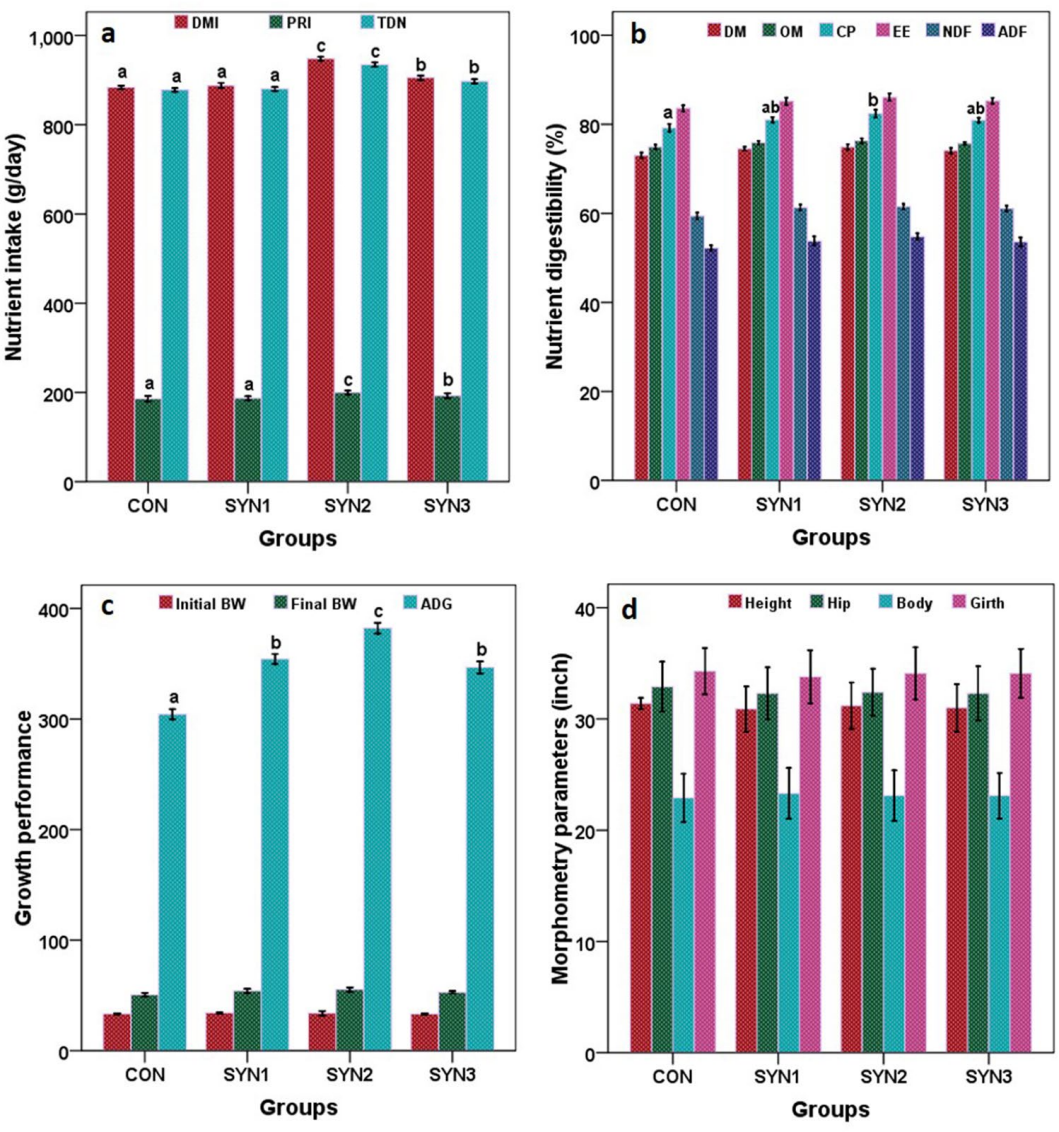
Nutrient intake, digestibility, growth performance and morphometry parameters in different groups of pre-ruminant buffalo calves (CON: Control), SYN1 (3 g FOS + *Lactobacillus plantarum* CRD7-100 ml), SYN2 (6 g FOS + *Lactobacillus plantarum* CRD7-150 ml) and SYN3 (9 g FOS + *Lactobacillus plantarum* CRD7-50 ml).

Dry matter intake, crude protein content and TDN intake were all affected by the synbiotics formulations, and SYN2 had the highest values (948.10, 199.90, 934.50 g/day), followed by SYN3 (905.40, 192.70, 897.30 g/day), SYN1 (887.90, 187.10, 880.20 g/day), and control (883.80, 186 and 877.90 g/day), respectively (Fig. 1a,b; Supplementary material 1).

The statistical analysis did not show significant effect (*P* > 0.05) of the addition of synbiotic formulations on the digestibility of organic matter and acid detergent fibre. Instead, supplementation altered (*P* < 0.05) crude protein digestibility and highest was found in SYN2 (82.40%), followed by SYN1 (81%), SYN3 (80.90%), and least was observed in control (79.20%), respectively. Although, there was no effect of the dietary addition of synbiotics on the digestibility of DM.

### Growth performance

All buffalo calves were in good and healthy condition prior to the start of the experimental trial. The initial average body weight was nearly same in all groups 33.50, 34.30, 34.0 and 33.80 kg in control, SYN1, SYN2 and SYN3, respectively. The final body weight average was higher in SYN2 (55.40 kg), followed by SYN1 (54.10 kg), SYN3 (52.80 kg), and CON (50.60 kg), respectively. Average daily gain (g) was also higher (*P* < 0.05) in SYN2 (382.20 g) followed by SYN1 (354.30 g), SYN3 (346.70 g), and the least was observed in the control (304.30 g) group (Fig. 1c).

### Skeletal morphometry

The height of calves was 31.40, 30.90, 31.20 and 31.00 inches in CON, SYN1, SYN2 and SYN3 groups, respectively. Hip height, body length and girth in calves were 32.90, 22.90 and 34.30 in control, 32.30, 23.30, 33.80 in SYN1, 32.40, 23.10, 34.10 in SYN2 and 32.30, 23.20 and 34.10 in SYN3, respectively. There were no changes (*P* > 0.05) in body, hip and girth length when different synbiotics formulations were provided to buffalo calves (Fig. 1d; SM2).

### Effect of synbiotics formulation on blood biochemical and haematological parameters in Buffalo calves

**Figure 2:**
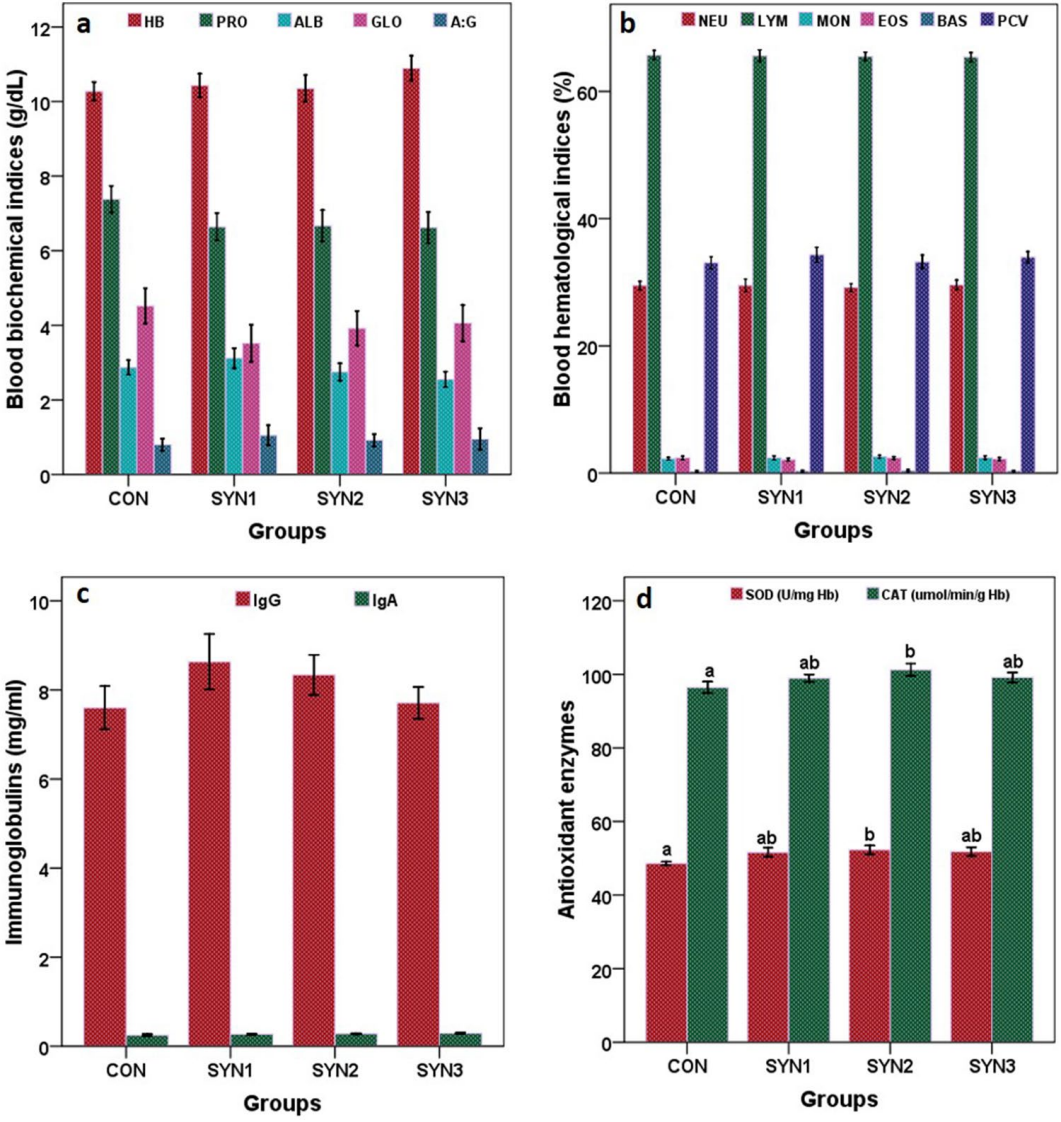
Effect of synbiotics formulation on blood biochemical and hematological indices, immunoglobulin and antioxidant enzyme activities in different groups of pre-ruminant buffalo calves (CON: Control), SYN1 (3 g FOS + *Lactobacillus plantarum* CRD7-100 ml), SYN2 (6 g FOS + *Lactobacillus plantarum* CRD7-150 ml) and SYN3 (9 g FOS + *Lactobacillus plantarum* CRD7-50 ml).

The general blood biochemical parameters including glucose (mg/dl), total protein (g/dl), albumin (g/dl) and A: G ratio, were presented in the supplementary material (Fig. 2a; SM3). The glucose concentration for the control, SYN1, SYN2, and SYN3 were 86.40, 83.60, 83.80 and 84.60 mg/dl, respectively. Total serum protein values in all groups remains unaffected, but found within biological range and showed 7.38, 6.64, 6.67 and 6.62 g/dl in control, SYN1, SYN2, and SYN3, respectively. Additionally, period-wise values remained consistent and none of the group’s serum globulin or A: G values changed.

Blood haematology indices including haemoglobin, PVC, TEC, and DLC of pre-ruminant buffalo calves were not significantly affected by the supplementation of synbiotics formulation (Fig. 2b; SM4, SM5). The average concentration of Hb for CON, SYN1, SYN2, and SYN3 were 10.27, 10.43, 10.35, and 10.89 g/dl, respectively, whereas PCV average for CON, SYN1, SYN2, and SYN3 were 33.07, 34.33, 33.22, and 33.94%, respectively. Synbiotics formulation had no impact on blood DLC.

### Immunoglobulins and antioxidant enzyme activities in buffalo calves

Synbiotics formulations had no effect on serum concentrations of both IgG and IgA (*P* > 0.05). IgG concentrations ranged from 7.60, 8.63, 8.34 and 7.71 mg/ml in control, SYN1, SYN2, and SYN3, while IgA concentration were 0.24, 0.26, 0.27 and 0.28 mg/ml in control, SYN1, SYN2 and SYN3, respectively (Fig. 2c).

SOD activity was higher in SYN2 (52.20 U/mg Hb) followed by SYN3 (51.80 U/mg Hb), and SYN1 (51.60 U/mg Hb) as compared to control (48.60 U/mg Hb). Catalase activity (µmol of H_2_O_2_) was also higher in SYN2 (101.23 µmol of H_2_O_2_) followed by SYN1 (98.95 µmol of H_2_O_2_), and SYN3 (99.16 µmol of H_2_O_2_), respectively (Fig. 2d; SM6).

### Humoral and cell mediated response in buffalo calves

**Figure 3:**
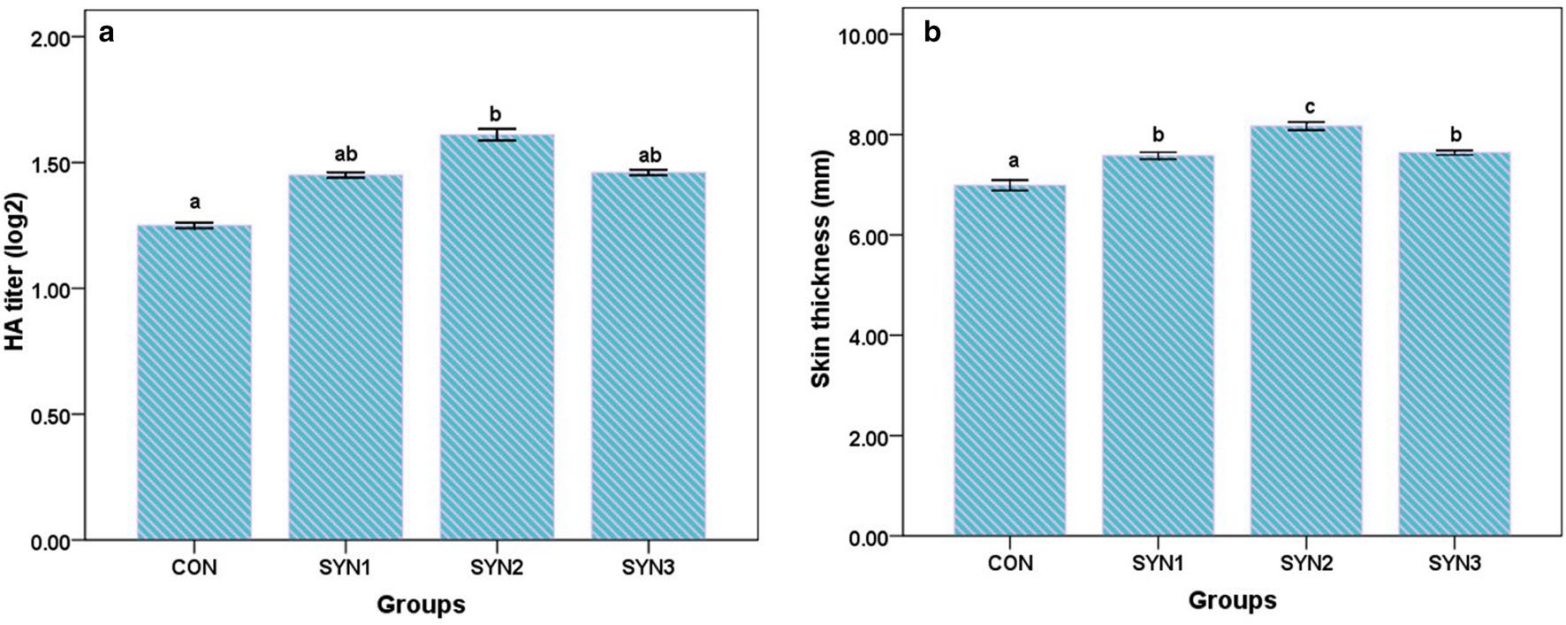
Effect of synbiotics formulation on pre-ruminant buffalo calves: (**a**) Humoral immunity and (**b**) Cell mediated immunity. CON (Control), SYN2 (6 g FOS + *Lactobacillus plantarum* CRD7-150 ml) and SYN3 (9 g FOS + *Lactobacillus plantarum* CRD7-50 ml).

The humoral immune response to chicken RBC followed an increment pattern until day fourteen, when it began to decline. The HMI average values were higher in all supplemented groups in comparison to control (Fig. 3a). The pattern was higher (*P* < 0.05) in SYN2 (2.16 and 1.61 mm) followed by SYN3 (1.91 and 1.46), SYN1 (1.87 and 1.45), and CON (1.63 and 1.25 HA log2), respectively on days fourteen and twenty-one. The CMI response was also higher in treated groups as compared to control at 28 and 48 h of recording. Skin thickness was greater in SYN2 (8.17 mm) followed by SYN3 (7.64 mm), SYN1 (7.58 mm), and control (6.99 mm), respectively (Fig. 3b).

### Faecal bacterial count, metabolites and VFA

The total viable count of *Lactobacillus* was higher in the SYN2 (9.15 cfu/g) followed by SYN3 (9.07 cfu/g), SYN1 (8.94 cfu/g), and least was observed in control group (8.77 cfu/g). Comparing the 60th day to 0th and 30th day, the faecal *Lactobacillus* count shows a positive change (*P* < 0.05). Total viable count of *Bifidobacterium* was also higher in SYN2 (9.22 cfu/g) followed by SYN3 (9.19 cfu/g), and SYN1 (9.17 cfu/g) as compared to control (8.69 cfu/g). On the other hand, total coliform and *Clostridium* count was higher (*P* < 0.05) in control as compared to treated groups (Table 1).

**Table 1:** Effect of synbiotics formulation on total viable bacterial count of feces (log 10 cfu/g) in different groups of pre-ruminant buffalo calves (CON: Control), SYN1 (3 g FOS + *Lactobacillus plantarum* CRD7-100 ml), SYN2 (6 g FOS + *Lactobacillus plantarum* CRD7-150 ml) and SYN3 (9 g FOS + *Lactobacillus plantarum* CRD7-50 ml).

Parameter	Dietary groups						
	CON	SYN1	SYN2	SYN3	T	*P* value	T*P
*Lactobacillus* (log_10_cfu/g)							
0 day	9.08 ± 0.14	8.68 ± 0.22	8.89 ± 0.08	8.92 ± 0.11	0.006	< 0.088	0.005
30 days	8.67 ± 0.19	8.94 ± 0.16	9.11 ± 0.10	9.10 ± 0.05			
60 days	8.55^a^ ± 0.14	9.20^b^ ± 0.20	9.44^b^ ± 0.11	9.20^b^ ± 0.06			
Average	8.77^a^ ± 0.11	8.94^ab^ ± 0.12	9.15^b^ ± 0.08	9.07^b^ ± 0.05			
*Bifidobacterium* (log_10_cfu/g)							
0 day	9.00 ± 0.06	8.96 ± 0.10	8.98 ± 0.04	8.91 ± 0.04	< 0.001	< 0.001	0.024
30 days	8.70^a^ ± 0.11	9.16^b^ ± 0.10	9.19^b^ ± 0.08	9.04^b^ ± 0.04			
60 days	8.36^a^ ± 0.15	9.38^b^ ± 0.16	9.50^b^ ± 0.09	9.62^b^ ± 0.11			
Average	8.69^a^ ± 0.09	9.17^b^ ± 0.08	9.22^b^ ± 0.07	9.19^b^ ± 0.08			
*Coliform* (log_10_cfu/g)							
0 day	8.97 ± 0.06	9.05 ± 0.08	8.96 ± 0.06	9.00 ± 0.07	0.001	< 0.001	0.039
30 days	8.93^b^ ± 0.06	8.86^ab^ ± 0.05	8.58^a^ ± 0.13	8.84^ab^ ± 0.07			
60 days	9.12^c^ ± 0.18	8.49^a^ ± 0.09	8.40^a^ ± 0.14	8.73^ab^ ± 0.14			
Average	9.01^b^ ± 0.07	8.80^ab^ ± 0.08	8.65^a^ ± 0.08	8.86^ab^ ± 0.06			
*Clostridia* (log_10_cfu/g)							
0 day	8.75 ± 0.10	8.94 ± 0.06	9.02 ± 0.12	8.85 ± 0.08	0.063	< 0.001	< 0.001
30 days	8.93 ± 0.11	8.61 ± 0.15	8.52 ± 0.13	8.62 ± 0.16			
60 days	8.87^b^ ± 0.08	8.36^a^ ± 0.10	8.37^a^ ± 012	8.30^a^ ± 0.17			
Average	8.85 ± 0.05	8.64 ± 0.09	8.63 ± 0.09	8.59 ± 0.09			

Means bearing different superscripts a and b in the same row differ significantly (*P* < 0.05).

The pH of faecal decreased (*P* < 0.05) in SYN1 (6.92), SYN2 (6.84), SYN3 (6.95), and higher was observed in control group (7.20). Opposite trend was observed for faecal lactic acid, SYN1, SYN2, and SYN3 showed 2.98, 3.30 and 2.89 µmol/g lactate and lower was observed in control (2.53 µmol/g). Ammonia concentration was found to be intermediate in SYN2 (5.98 µmol/g) followed by SYN1 (6.11 µmol/g), SYN3 (6.25 µmol/g), and control (6.63 µmol/g) (Table 2).

**Table 2:** Effect of synbiotics bioformulation on fecal metabolites and pH in different groups of pre-ruminant buffalo calves (CON: Control), SYN1 (3 g FOS + *Lactobacillus plantarum* CRD7-100 ml), SYN2 (6 g FOS + *Lactobacillus plantarum* CRD7-150 ml) and SYN3 (9 g FOS + *Lactobacillus plantarum* CRD7-50 ml).

Parameters	Dietary groups						
	CON	SYN1	SYN2	SYN3	T	*P* value	T*P
Lactate (µmol/g)							
0 day	2.49 ± 0.29	2.73 ± 0.20	2.68 ± 0.14	2.58 ± 0.18	0.005	0.013	0.643
30 days	2.54^a^ ± 0.17	3.07^ab^ ± 0.24	3.54^b^ ± 0.07	3.00^ab^ ± 0.36			
60 days	2.56 ± 0.25	3.15 ± 0.14	3.68 ± 0.38	3.09 ± 0.29			
Average	2.53^a^ ± 0.13	2.98^ab^ ± 0.12	3.30^b^ ± 0.17	2.89^ab^ ± 0.16			
Ammonia (µmol/g)							
0 day	6.59 ± 0.21	6.29 ± 0.18	6.46 ± 0.21	6.63 ± 0.31	0.018	0.016	0.662
30 days	6.73 ± 0.23	6.24 ± 0.41	5.88 ± 0.21	6.24 ± 0.21			
60 days	6.57^b^ ± 0.39	5.81^ab^ ± 0.22	5.61^a^ ± 0.17	5.88^ab^ ± 0.15			
Average	6.63^b^ ± 0.15	6.11^a^ ± 0.17	5.98^a^ ± 0.14	6.25^ab^ ± 0.15			
pH							
0 day	7.08 ± 0.10	7.28 ± 0.18	7.12 ± 0.14	7.18 ± 0.08	0.037	0.001	0.198
30 days	7.33 ± 0.19	6.85 ± 0.16	6.87 ± 0.13	7.03 ± 0.18			
60 days	7.19^b^ ± 0.10	6.63^ab^ ± 0.19	6.53^a^ ± 0.14	6.65^ab^ ± 0.18			
Average	7.20^b^ ± 0.08	6.92^ab^ ± 0.12	6.84^a^ ± 0.09	6.95^ab^ ± 0.10			

Means bearing different superscripts a and b in the same row differ significantly (*P* < 0.05).

Synbiotic formulations had higher (*P* < 0.05) levels of acetate and butyrate in SYN2 (17.70 and 4.76 µmol/g) followed by SYN1 (17.70 and 4.76 µmol/g), and SYN3 (17.50 and 4.43 µmol/g) as compared to control group (16.40 and 3.87 µmol/g), but had no effect on the acetate to propionate ratio in the faecal samples (Table 3).

**Table 3:** Effect of synbiotics bioformulation on fecal volatile fatty acids in different groups of pre-ruminant buffalo calves (CON: Control), SYN1 (3 g FOS + *Lactobacillus plantarum* CRD7-100 ml), SYN2 (6 g FOS + *Lactobacillus plantarum* CRD7-150 ml) and SYN3 (9 g FOS + *Lactobacillus plantarum* CRD7-50 ml).

Parameters	Dietary groups						
	CON	SYN1	SYN2	SYN3	T	*P*	T*P
Acetate (µmol/g)							
0 day	15.60 ± 1.27	16.30 ± 0.74	16.50 ± 0.44	15.90 ± 0.70	0.015	< 0.001	0.883
30 days	16.60 ± 0.65	18.10 ± 0.92	18.70 ± 0.57	17.90 ± 0.79			
60 days	17.10^a^ ± 0.57	18.80^ab^ ± 0.46	20.10^ab^ ± 0.59	18.80^b^ ± 0.77			
Average	16.40^a^ ± 0.50	17.70^ab^ ± 0.48	18.40^b^ ± 0.47	17.50^ab^ ± 0.50			
Propionate (µmol/g)							
0 day	7.87 ± 0.53	8.05 ± 0.48	8.06 ± 0.41	7.46 ± 0.31	0.060	0.034	0.573
30 days	8.06 ± 0.50	8.57 ± 0.36	8.85 ± 0.49	8.45 ± 0.19			
60 days	7.59^a^ ± 0.25	8.59^ab^ ± 0.24	9.25^b^ ± 0.48	8.68^ab^ ± 0.29			
Average	7.84 ± 0.24	8.40 ± 0.21	8.72 ± 0.28	8.20 ± 0.19			
Butyrate (µmol/g)							
0 day	3.55 ± 0.22	4.07 ± 0.34	3.77 ± 0.34	3.59 ± 0.36	< 0.001	< 0.001	0.311
30 days	3.85^a^ ± 0.27	4.87^ab^ ± 0.25	5.29^b^ ± 0.32	4.76^ab^ ± 0.21			
60 days	4.22^a^ ± 0.28	5.35^ab^ ± 0.28	6.04^b^ ± 0.50	4.96^ab^ ± 0.27			
Average	3.87^a^ ± 0.16	4.76^b^ ± 0.21	5.03^c^ ± 0.31	4.43^b^ ± 0.21			
A:P							
0 day	2.05 ± 0.27	2.05 ± 0.17	2.08 ± 0.14	2.16 ± 0.14	0.998	0.485	0.997
30 days	2.11 ± 0.19	2.13 ± 0.15	2.15 ± 0.16	2.12 ± 0.12			
60 days	2.26 ± 0.14	2.20 ± 0.09	2.22 ± 0.16	2.17 ± 0.09			
Average	2.14 ± 0.11	2.13 ± 0.08	2.15 ± 0.08	2.15 ± 0.06			

Means bearing different superscripts a and b in the same row differ significantly (*P* < 0.05).

### Diarrheal incidence and faecal score

**Figure 4:**
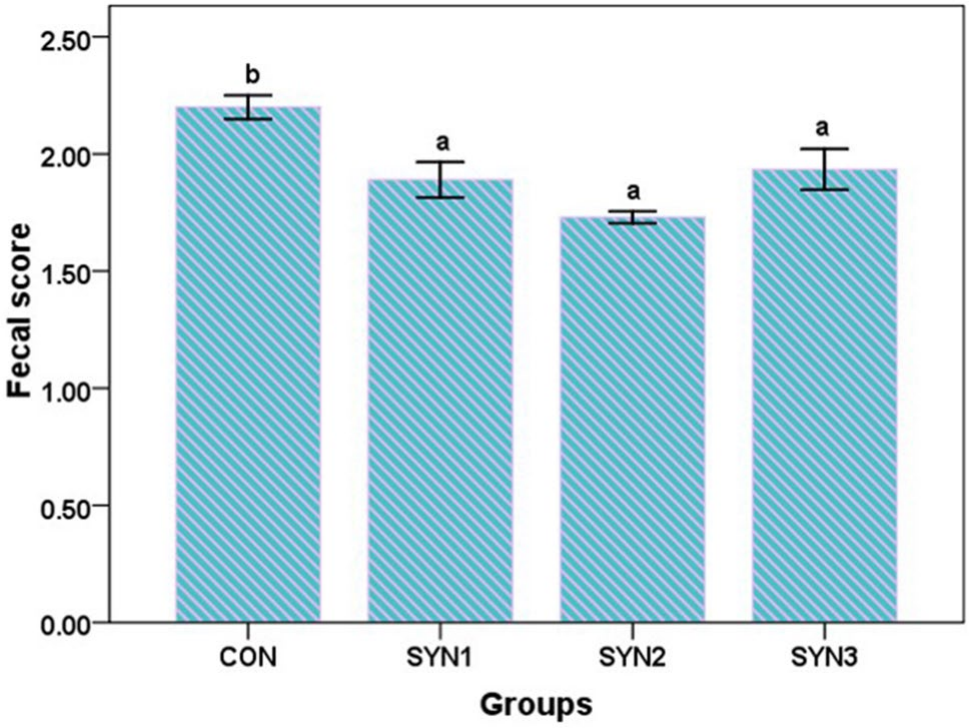
Effect of synbiotics formulation on fecal score in different groups of pre-ruminant buffalo calves; (CON: Control), SYN1 (3 g FOS + *Lactobacillus plantarum* CRD7-100 ml), SYN2 (6 g FOS + *Lactobacillus plantarum* CRD7-150 ml) and SYN3 (9 g FOS + *Lactobacillus plantarum* CRD7-50 ml).

The average faecal score was lower in all supplemented groups with SYN1 (1.89), SYN2 (1.73) and SYN3 (1.93) values lower than in the control group (2.20). In this study we observed that synbiotics treatment resulted significant reduction in faecal score. During the first week of the experiment, one calf in each group had diarrhoea, however as the experiment progressed, the incidence of diarrhoea in supplemented groups decreased in comparison to control group (Fig. 4).

## Discussion

Dairy farms must manage proper nutrition and feeding management for their calves to ensure long-term financial success. Synbiotic supplementation is a crucial tool for improving growth and reduce the negative consequences of raising calves. Synbiotics are well-known for their beneficial effect on feed consumption, growth performance, and gastrointestinal health^37^. Our findings revealed a beneficial effect on nutrient intake, protein digestibility, and NDF. This may be due to the positive effects of synbiotics formulations on gut health. Lucey et al.^38^ reported that mannan-oligosaccharide and *Bacillus subtilis* formulation improved ADG, and potential health benefits in calves. The *Saccharomyces cerevisiae* feeding improves nutrient digestibility in cattle by balancing the rumen environment^39^. The prebiotics/probiotics together improved digestion and ruminal fermentation in lambs fed high-energy diets as reported by Zapata et al.^40^.

The results of the current study revealed that synbiotics formulations had a considerable impact on calve’s daily weight gain due to the positive effect of synbiotics on beneficial microbiota of GI which reflects more absorption of nutrients thus facilitated better growth rate. According to Estrada-Angulo^41^, supplementation of prebiotics and probiotics increased dietary energy efficiency under subtropical climate in lambs. The addition of Eubiotic feed supplements increased average daily gain, ruminal fermentation, and lowered faecal scores^42^. Zhang et al.^43^ reported that *Lactobacillus rhamnosus* supplementation in calves fed improved growth performance, total VFA, butyrate, and propionate concentration in rumen fluids of the treated neonatal calves as compared to control group due to the modulation of rumen and intestinal microbe balance. Presence of higher concentration of VFA indicates that probiotics are adapting well in gut and are positively utilising prebiotics. Moarrab et al.^44^ observed that using synbiotics improved growth performance, faecal microbes and blood metabolites due to improvement of diet digestibility and feed efficiency in sucking lamb.

Body length and hip height are bone growth parameters, while chest size is a measure of muscle, bone and fat development that is proportional to live body weight. In this study, synbiotics formulations had no effect on the morphometry of pre-ruminant buffalo calves. However, Sharma et al.^36^ found positive effect of prebiotics and probiotics on hip height and heart girth due to increased nutrient and energy availability and DM intake.

The most commonly used diagnostic decision-making methods are haematological parameters which are extremely useful in determining an animal metabolism and their health status. This study found that synbiotics supplementation had no effect on any blood biochemical and haematological parameters. However, application of *Bacillus licheniformis* and *B. subtilis* increased total protein, albumin, globulins content, improve intestinal microflora, and immunity status in sheep and lambs^45^. The observed disparity in blood parameters could be attributed to variations in probiotics and prebiotics source, as well as climate variables and basal nutrient level.

The antioxidant enzyme activities in the current study were higher, which could be attributed to the combined impact of prebiotics and probiotics. Probiotic can boost antioxidant activity because they have their own antioxidant system which includes enzymes such as CAT and SOD that stimulate the antioxidant signalling pathway^46^. Furthermore, prebiotics and probiotics have the ability to strengthen the hosts own antioxidant system and increase its activity. MOS supplementation in sheep diet enhanced SOD and CAT activities as compared to control group^47^. Probiotics feeding could boost antioxidant potential and immune response by balancing the intestinal microbiota in buffalo calves^48^.

Calves with naive immune system come into contact to environmental factors which coincides with microbial colonization thus this period is critical for proper development of GIT microbiota and ultimately, a functional immune system. Probiotic bacteria may help by competitively excluding undesirable microbes, interact with gut epithelial cells and have immunomodulatory effects^49^. *Bacillus-*based electrolyte containing fed supplement promotes the activation of T cell and mature cells such as CD8(−) CD25(+) and CD8(−) TCRs in dairy calves^50^. Repeated administration of probiotics may boost cellular immunity and aid recovery from scouring^51^. In this study, there was no change in IgA and IgG levels. Supplementation of *S. cerevisiae* improved blood immunoglobins content (IgA, IgG and IgM), modulated rectal microbiota, and decreased the incidence of diarrhoea in young dairy calves^52^.

We found that supplemented groups had lower faecal pH and higher levels of lactate, acetate, and butyrate in comparison to control group due to the production of metabolites. Lactate and butyrate producing bacteria aids in the integrity and development of rumen and intestinal epithelium. The production of VFAs reduces the population of harmful bacteria. Probiotics produce antimicrobial and metabolic compounds, and they can lower ruminal pH by secreting acetic and lactic acid, which inhibits the growth of pathogens that are responsible for poor faecal health^53^.

The balance of gastrointestinal microbiota has a significant impact on digestion, nutrient absorption and animal health. In the current study, CFU count of *Lactobacillus* and *Bifidobacterium* in all synbiotics groups were higher than control resulting higher lactate concentration in faeces. It is well known that the prebiotic inulin promotes the growth of lactic acid bacteria such as *Bifidobacterium* and *Lactobacillus,* which has a favourable impact on microflora and increases nutrient availability and absorption^54^. Feeding lactic acid bacteria and yeast, augmented the richness of useful bacteria such as *Bifidobacterium* in the intestinal tract of new-born calves revealed using metagenomic approach^55^. Probiotics influenced the gut microbiota by reduce the abundance of *Desulfovibrio* while increase the abundance of *Lactobacillus* and *Bifidobacterium* reported by Wang et al.^56^.

Neonatal diarrhoea is the most common reason of death in calves, results in a significant financial loss for dairy farms around the world. In this study supplementation of prebiotics and probiotics reduced diarrhoea incidence due to decrease in the count of pathogenic bacterial population of *Clostridium* and total coliform, provide favourable environmental conditions, and growth factors for GI beneficial bacteria, resulting in an increase in total *lactobacilli*^57^. Combination of *L. plantarum* and *B. subtilis* in the feed can help balance the intestinal flora and prevent calf diarrhoea^58^. According to Kawakami et al.^59^, giving calves milk substitutes incorporating yeast and lactic acid bacteria substantially increased their feed conversion ratio, decreased faecal score, and prevented diarrhoea in calves. Lactic acid bacteria can manage the intestinal flora balance in calves, decrease the pathogenic bacteria in faeces, improve their health status and minimize diarrhoea^60^. Synbiotics may also help to improve gut immunity, and the mucosal barrier system in the gut, which is the first defensive mechanism and may help to reduce diarrhoea. Supplementation of Inulin and *L. casei* gained weight, and improved intestinal health in dorset lambs by lowering coliforms and diarrhoea incidence^57^. Prebiotics and probiotics may have a beneficial effect by boosting immune system, reducing stress and diarrhoea prevention.

In conclusion, the findings of this study indicate that combination of 6 g fructo-oligosaccharides and *L. plantarum* CRD-7 (100 ml) as fermented milk was beneficial in terms of average daily gain, digestibility, faecal microbiota and metabolites, antioxidant activity, and reduced faecal score and diarrhoea incidence. Additionally, pre-ruminant buffalo calves showed improvements in humoral and cell-mediated immunity. However, further research is required to fully understand the fundamental aspects of future synbiotics research on structure and gut microbiota, as well as host-microbe relationship. Similarly, there is a need to investigate the feasibility of developing a commercial synbiotic formulation for use as prophylaxis in pre-ruminant calves.

## Supplementary Information

Supplementary Information.

## Data Availability

The study material related to this study are included in this article and supplementary material.

## References

[1] Amin N, Seifert J (2021). Dynamic progression of the calf's microbiome and its influence on host health. Comput. Struct. Biotechnol. J..

[2] Uyeno Y, Shigemori S, Shimosato T (2015). Effect of probiotics/prebiotics on cattle health and productivity. Microbes Environ..

[3] Caffarena RD, Casaux ML, Schild CO, Fraga M, Castells M, Colina R, Maya L, Corbellini LG, Riet-Correa F, Giannitti F (2021). Causes of neonatal calf diarrhea and mortality in pasture-based dairy herds in Uruguay: A farm-matched case-control study. Braz. J. Microbiol..

[4] Andremont A, Cervesi J, Bandinelli PA, Vitry F, Gunzburg JD (2021). Spare and repair the gut microbiota from antibiotic-induced dysbiosis: State-of-the-art. Drug Discov. Today.

[5] Azad M, Gao J, Li T, Tan B, Huang X, Yin J (2020). Opportunities of prebiotics for the intestinal health of monogastric animals. Anim. Nutr..

[6] Markowiak P, Slizewska K (2017). Effects of probiotics, prebiotics, and synbiotics on human health. Nutrients.

[7] Cangiano LR, Yohe TT, Steele MA, Renaud DL (2020). Invited review: Strategic use of microbial-based probiotics and prebiotics in dairy calf rearing. Appl. Anim. Sci..

[8] Hasunuma T, Kawashima K, Nakayama H, Murakami T, Kanagawa H, Ishii T, Akiyama K, Yasuda K, Terada F, Kushibiki S (2011). Effect of cellooligosaccharide or synbiotic feeding on growth performance, fecal condition and hormone concentrations in holstein calves. Anim. Sci. J..

[9] Li Z, Bai H, Zheng L, Jiang H, Cui H, Cao Y, Yao J (2018). Bioactive polysaccharides and oligosaccharides as possible feed additives to manipulate rumen fermentation in Rusitec fermenters. Int. J. Biol. Macromol..

[10] Chang M, Wang F, Ma F, Jin Y, Sun P (2022). Supplementation with galacto-oligosaccharides in early life persistently facilitates the microbial colonization of the rumen and promotes growth of preweaning Holstein dairy calves. Anim. Nutr..

[11] Berge AC (2016). A meta-analysis of the inclusion of Bio-Mos® in milk or milk replacer fed to dairy calves on daily weight gain in the pre-weaning period. J. Anim. Res. Nutr..

[12] Bidarkar VK, Swain PS, Ray S, Dominic G (2014). Probiotics: Potential alternative to antibiotics in ruminant feeding. Trends Vet. Anim. Sci..

[13] Sato N, Garcia-Castillo V, Yuzawa M, Islam MA, Albarracin L, Tomokiyo M, Ikeda-Ohtsubo W, Garcia-Cancino A, Takahashi H, Villena J (2020). Immunobiotic *Lactobacillus jensenii* TL2937 alleviates dextran sodium sulfate-induced colitis by differentially modulating the transcriptomic response of intestinal epithelial cells. Front. Immunol..

[14] Kober AKMH, Riaz Rajoka MS, Mehwish HM, Villena J, Kitazawa H (2022). Immunomodulation potential of probiotics: A novel strategy for improving livestock health, immunity, and productivity. Microorganisms.

[15] Zhang R, Zhou M, Tu Y, Zhang NF, Deng KD, Ma T, Diao QY (2016). Effect of oral administration of probiotics on growth performance, apparent nutrient digestibility and stress-related indicators in Holstein calves. J. Anim. Physiol. Anim. Nutr..

[16] Khaziakhmetov F, Khabirov A, Tagirov K, Avzalov R, Tsapalova G, Basharov A (2020). The influence of “Stimix Zoostim” and “Normosil” probiotics on fecal microflora, hematologic indicators, nutrient digestibility, and growth of mother-bonded calves. Vet. World.

[17] Wu Y, Wang L, Luo R, Chen H, Nie C, Niu J, Chen C, Xu Y, Li X, Zhang W (2021). Effect of a multispecies probiotic mixture on the growth and incidence of diarrhea, immune function, and fecal microbiota of pre-weaning dairy calves. Front. Microbiol..

[18] Wang H, Yu Z, Gao Z, Li Q, Qiu X, Wu F, Guan T, Cao B, Su H (2022). Effects of compound probiotics on growth performance, rumen fermentation, blood parameters, and health status of neonatal Holstein calves. J. Dairy Sci..

[19] Bayatkouhsar J, Tahmasebi AM, Naserian AA, Mokarram RR, Valizadeh R (2013). Effects of supplementation of lactic acid bacteria on growth performance, blood metabolites and fecal coliform and lactobacilli of young dairy calves. Anim. Feed Sci. Technol..

[20] Ngo TT, Bang NN, Dart P, Callaghan M, Klieve A, Hayes B, McNeill D (2021). Feed preference response of weaner bull calves to *Bacillus amyloliquefaciens* H57 probiotic and associated volatile organic compounds in high concentrate feed pellets. Animals.

[21] Villot C, Chen Y, Pedgerachny K, Chaucheyras-Durand F, Chevaux E, Skidmore A (2020). Early supplementation of *Saccharomyces cerevisiae boulardii* CNCM I-1079 in newborn dairy calves increases IgA production in the intestine at 1 week of age. J. Dairy Sci..

[22] Arne A, Ilgaza A (2021). Prebiotic and synbiotic effect on rumen papilla length development and rumen pH in 12-week-old calves. Vet. World.

[23] Trukhachev VI, Buryakov NP, Shapovalov SO, Shvydkov AN, Buryakova MA, Khardik IV, Fathala MM, Komarova OE, Aleshin DE (2022). Impact of inclusion of multicomponent synbiotic Russian Holstein dairy cow's rations on milk yield, rumen fermentation, and some blood biochemical parameters. Front. Vet. Sci..

[24] Jonova S, Ilgaza A, Zolovs M (2021). The impact of inulin and a novel synbiotic (yeast *Saccharomyces cerevisiae* strain 1026 and inulin) on the development and functional state of the gastrointestinal canal of calves. Vet. Med. Int..

[25] ICAR (2013). Nutrient Requirements of Cattle and Buffalo.

[26] Van Soest PJ, Robertson JB, Lewis BA (1991). Methods for dietary fiber, neutral detergent fiber, and nonstarch polysaccharides in relation to animal nutrition. J. Dairy Sci..

[27] AOAC (2005). Official Methods of Analysis. Association of Official Analytical Chemist.

[28] Henry RJ (1964). Clinical Chemistry-Principles and Techniques.

[29] Gornall AG, Barsawill CJ, David MM (1949). Determination of serum proteins by means of the biuret reaction. J. Biol. Chem..

[30] Gustaffson EJ (1978). Improved specificity of serum albumin determination and estimation of acute phase reactant by use of the bromocresol green reduction. Clin. Chem..

[31] Madhesh M, Balsubramaniam KA (1998). Micro titer plate assay for superoxide dismutase using MTT reduction by superoxide. Ind. J. Biochem. Biophys..

[32] Aebi H (1994). Catalase in vitro. Methods Enzymol..

[33] Pattanaik AK, Khan SA, Goswami TK (2011). Iodine supplementation to a diet containing *Leucaena leucocephala* leaf meal: Consequences on nutrient metabolism, clinical chemistry and immunity of goats. Anim. Prod. Sci..

[34] Masucci F, De Rosa G, Grasso F, Napolitano F, Esposito G (2011). Performance of immune response of buffalo calves supplemented with probiotic. Livest. Sci..

[35] Sharma AN, Kumari LV, Ram C, Mondal G (2020). Response of different synbiotics on gut health, immunity and growth performance of pre-ruminant Buffalo calves. Indian J. Anim. Nutr..

[36] Sharma AN, Kumar S, Tyagi AK (2018). Effects of mannan-oligosaccharides and *Lactobacillus acidophilus* supplementation on growth performance, nutrient utilization and faecal characteristics in Murrah buffalo calves. J. Anim. Physiol. Anim. Nutr..

[37] Gomez Quintero DF, Kok CR, Hutkins R (2022). The future of synbiotics: Rational formulation and design. Front. Microbiol..

[38] Lucey PM, Lean IJ, Aly SS, Golder HM, Block E, Thompson JS, Rossow HA (2021). Effects of mannan-oligosaccharide and *Bacillus subtilis* supplementation to preweaning Holstein dairy heifers on body weight gain, diarrhea, and shedding of fecal pathogens. J. Dairy Sci..

[39] Anjum MI, Javaid S, Ansar MS, Ghaffar A (2018). Effects of yeast (*Saccharomyces cerevisiae*) supplementation on intake, digestibility, rumen fermentation and milk yield in Nili-Ravi buffaloes. Iran. J. Vet. Res..

[40] Zapata O, Cervantes A, Barreras A, Monge-Navarro F, González-Vizcarra VM, Estrada-Angulo A, Urías-Estrada JD, Corono L, Zinn RA, Martínez-Alvarez IG (2021). Effects of single or combined supplementation of probiotics and prebiotics on ruminal fermentation, ruminal bacteria and total tract digestion in lambs. Small Rumin. Res..

[41] Estrada-Angulo A, Zapata-Ramírez O, Castro-Pérez BI, Urías-Estrada JD, Gaxiola-Camacho S, Angulo-Montoya C, Ríos-Rincón FG, Barreras A, Zinn RA, Leyva-Morales JB (2021). The effects of single or combined supplementation of probiotics and prebiotics on growth performance, dietary energetics, carcass traits, and visceral mass in lambs finished under subtropical climate conditions. Biology.

[42] Stefańska B, Katzer F, Golińska B, Sobolewska P, Smulski S, Frankiewicz A, Nowak W (2022). Different methods of eubiotic feed additive provision affect the health, performance, fermentation, and metabolic status of dairy calves during the preweaning period. BMC Vet. Res..

[43] Zhang L, Jiang X, Liu X, Zhao X, Liu S, Li Y, Zhang Y (2019). Growth, health, rumen fermentation, and bacterial community of Holstein calves fed *Lactobacillus rhamnosus* GG during the preweaning stage1. J. Anim. Sci..

[44] Moarrab A, Ghoorchi T, Ramezanpour S, Ganji F, Koochakzadeh AR (2016). Effect of synbiotic on peformance, intestinal morphology, fecal microbial population and blood metabolites of suckling lambs. Iran. J. Appl. Anim. Sci..

[45] Devyatkin V, Mishurov A, Kolodina E (2021). Probiotic effect of *Bacillus subtilis* B-2998D, B-3057D, and *Bacillus licheniformis* B-2999D complex on sheep and lambs. J. Adv. Vet. Anim. Res..

[46] Wang Y, Wu Y, Wang Y, Xu H, Mei X, Yu D, Wang Y, Li W (2017). Antioxidant properties of probiotic bacteria. Nutrients.

[47] Zheng C, Li F, Hao Z, Liu T (2018). Effects of adding mannan oligosaccharides on digestibility and metabolism of nutrients, ruminal fermentation parameters, immunity, and antioxidant capacity of sheep. J. Anim. Sci..

[48] Varada VV, Kumar S, Tyagi N, Tyagi AK (2022). Effects of compound lyophilized probiotics on selected faecal microbiota, immune response, and antioxidant status in newborn buffalo calves. Curr. Res. Biotechnol..

[49] Kawakami S, Tomoya Y, Naota N (2010). Leukocyte phagocytic activity with or without probiotics in Holstein calves. Res. J. Biol. Sci..

[50] Novak KN, Davis E, Wehnes CA, Shields DR, Coalson JA, Smith AH, Rehberger TG (2012). Effect of supplementation with an electrolyte containing a *Bacillus*-based direct-fed microbial on immune development in dairy calves. Res. Vet. Sci..

[51] Qadis AQ, Goya S, Yatsu M, Kimura A, Ichijo T, Sato S (2014). Immune-stimulatory effects of a bacteria-based probiotic on peripheral leukocyte subpopulations and cytokine mRNA expression levels in scouring holstein calves. J. Vet. Med. Sci..

[52] Lu Q, Niu J, Wu Y, Zhang W (2022). Effects of *Saccharomyces*
*cerevisiae* var. boulardii on growth, incidence of diarrhea, serum immunoglobulins, and rectal microbiota of suckling dairy calves. Livest. Sci..

[53] Messaoudi S, Manai M, Kergourlay G (2013). Review *Lactobacillus salivarius*: Bacteriocin and probiotic activity. Food Microbiol..

[54] Singh AK, Kerketta S, Yogi RK, Kumar A, Ojha L (2017). Prebiotics: The new feed supplement for dairy calf. Int. J. Livest. Res..

[55] Liu B, Wang C, Huasai S, Han A, Zhang J, He L, Aorigele C (2020). Compound probiotics improve the diarrhea rate and intestinal microbiota of newborn calves. Animals.

[56] Wang T, Yan H, Lu Y, Li X, Wang X, Shan Y (2020). Anti-obesity effect of *LactoBacillus rhamnosus* LS-8 and *LactoBacillus crustorum* MN047 on high-fat and high-fructose diet mice base on inflammatory response alleviation and gut microbiota regulation. Eur. J. Nutr..

[57] Ayala-Monter MA, Hernández-Sánchez D, González-Muñoz S, Pinto-Ruiz R, Martínez-Aispuro JA, Torres-Salado N, Herrera-Pérez J, Gloria-Trujillo A (2019). Growth performance and health of nursing lambs supplemented with inulin and *Lactobacillus casei*. Asian Australas. J. Anim. Sci..

[58] Lee YE, Kang IJ, Yu EA, Kim S, Lee HJ (2012). Effect of feeding the combination with *Lactobacillus plantarum* and *Bacillus subtilis* on fecal microflora and diarrhea incidence of Korean native calves. Korean J. Vet. Serv..

[59] Kawakami SI, Yamada T, Nakanishi N, Cai Y (2011). Feeding of lactic acid bacteria and yeast affects fecal flora of Holstein calves. J. Anim. Vet. Adv..

[60] An HM, Lee DK, Cha MK, Lee SW, Lee SJ, Kim BS, Ha NJ (2011). Effects of lactic acid bacteria (LAB) supplement on the growth rate and elimination of enteropathogenic bacteria in calves. Biotechnol. Biotechnol. Equip..

