# Supplementary material for: Effect of synbiotics on growth performance, gut health, and immunity status in pre-ruminant buffalo calves
Source: Sci Rep. 2023 Jun 22;13:10184. doi: 10.1038/s41598-023-37002-6 (PMC10287688; doi:10.1038/s41598-023-37002-6)
Supplement: Supplementary file 1 — Supplementary Information. [file 41598_2023_37002_MOESM1_ESM.docx]

**Supplementary Material**

**SM1:** Growth performance, nutrient intake and utilization in different groups of pre-ruminant buffalo calves (CON: Control), SYN1 (3g FOS+ *Lactobacillus plantarum* CRD7-100ml), SYN2 (6g FOS+ *Lactobacillus plantarum* CRD7-150ml) and SYN3 (9g FOS+ *Lactobacillus plantarum* CRD7-50ml)

| **Parameters** | | **Dietary groups** | | | |  |
| --- | --- | --- | --- | --- | --- | --- |
|  |  | **CON** | **SYN1** | **SYN2** | **SYN3** | ***P*-value** |
| **Growth performance** | |  |  |  |  |  |
| Dry matter intake (g/day) | 883.80^a^±1.95 | | 887.90^a^±2.96 | 948.10^c^±2.10 | 905.40^b^±2.41 | 0.030 |
| Protein intake (g/day) | 186.00^a^±3.00 | | 187.10^a^±2.10 | 199.90^c^±2.09 | 192.70^b^±2.71 | 0.040 |
| TDN intake (g/day) | 877.90^a^±2.09 | | 880.20^a^±2.20 | 934.50^c^±2.47 | 897.30^b^±2.30 | 0.045 |
| Initial body weight (kg) | 33.50±0.53 | | 34.30±0.55 | 34.00±0.50 | 33.30±0.54 | 0.576 |
| Final body weight (kg) | 50.60±1.49 | | 54.10±1.61 | 55.40±1.74 | 52.80±0.96 | 0.161 |
| Average daily gain (g) | 304.30^a^±3.99 | | 354.30^b^±3.97 | 382.20^c^±4.70 | 346.70^b^±4.20 | 0.040 |
| **Apparent nutrient digestibility (%)** |  | |  |  |  |  |
| Dry matter | 73.00±0.55 | | 74.50±0.36 | 74.90±0.53 | 74.10±0.48 | 0.080 |
| Organic matter | 74.90±0.42 | | 75.90±0.33 | 76.30±0.47 | 75.70±0.27 | 0.112 |
| Crude protein | 79.20^a^±0.75 | | 81.00^ab^±0.50 | 82.40^b^±0.77 | 80.90^ab^±0.46 | 0.022 |
| Ether extract | 83.60±0.57 | | 85.20±0.68 | 86.10±0.71 | 85.30±0.57 | 0.078 |
| Neutral detergent fiber | 59.40±0.65 | | 61.30±0.58 | 61.50±0.54 | 61.10±0.53 | 0.065 |
| Acid detergent fiber | 52.20±0.52 | | 53.80±0.89 | 54.80±0.62 | 53.60±0.86 | 0.149 |

Means bearing different superscripts a, b and c in the same row differ significantly (*P*<0.05)

**SM2:** Effect of synbiotics formulation on morphometry in different groups of pre-ruminant buffalo calves (CON: Control), SYN1 (3g FOS+ *Lactobacillus plantarum* CRD7-100ml), SYN2 (6g FOS+ *Lactobacillus plantarum* CRD7-150ml) and SYN3 (9g FOS+ *Lactobacillus plantarum* CRD7-50ml)

| **Parameter**  **(Inch)** | **Dietary groups** | | | |  |  |  |
| --- | --- | --- | --- | --- | --- | --- | --- |
|  | **CON** | **SYN1** | **SYN2** | **SYN3** | **T** | ***P*-value (Linear/Quadratic)** | **T*P**  **(Linear/Quadratic)** |
| **Height** |  |  |  |  |  |  |  |
| 0 day | 29.80±0.40 | 29.20±0.91 | 29.60±1.16 | 29.20±0.97 | 0.408 | <0.001/004 | <0.001/0.882 |
| 30 days | 31.30±0.34 | 30.70±1.17 | 31.00±1.30 | 30.90±1.21 |  |  |  |
| 60 days | 33.20±0.26 | 32.70±0.98 | 33.00±1.05 | 32.90±1.04 |  |  |  |
| Average | 31.40±0.42 | 30.90±1.75 | 31.20±1.80 | 31.00±1.85 |  |  |  |
| **Hip Height**  0 day | 30.70±0.89 | 30.20±1.01 | 30.50±1.09 | 30.10±0.90 | 0.313 | <0.001/0.001 | <0.001/0.001 |
| 30 days | 33.00±0.77 | 32.30±0.87 | 32.70±1.19 | 32.30±0.88 |  |  |  |
| 60 days | 34.90±0.67 | 34.50±0.88 | 34.00±0.98 | 34.40±1.39 |  |  |  |
| Average | 32.90±1.94 | 32.30±2.02 | 32.40±1.82 | 32.30±2.10 |  |  |  |
| **Body length**  0 day | 21.50±1.45 | 21.80±1.54 | 21.80±1.71 | 21.50±1.08 | 0.915 | <0.001/0.635 | 0.819/0.954 |
| 30 days | 22.90±1.79 | 23.40±1.72 | 23.10±1.83 | 23.00±1.16 |  |  |  |
| 60 days | 24.50±1.12 | 24.90±1.52 | 24.50±1.60 | 24.80±1.36 |  |  |  |
| Average | 22.90±1.87 | 23.30±1.98 | 23.10±1.97 | 23.10±1.77 |  |  |  |
| **Girth** |  |  |  |  |  |  |  |
| 0 day | 32.40±0.61 | 32.10±1.41 | 31.70±0.63 | 32.00±0.76 | 0.699 | < 0.001/1.00 | <0.001/1.00 |
| 30 days | 34.20±0.84 | 33.60±1.44 | 34.30±0.81 | 34.10±0.92 |  |  |  |
| 60 days | 36.20±0.99 | 35.70±1.60 | 36.20±0.86 | 36.10±0.92 |  |  |  |
| Average | 34.30±1.80 | 33.80±2.06 | 34.10±2.03 | 34.10±1.91 |  |  |  |

**SM3:** Effect of synbiotics formulation on blood biochemical indices of pre-ruminant buffalo calves after different time interval

| **Parameter** | | **Dietary groups** | | | | | | | **T** | **P** | **T*P** |  | |
| --- | --- | --- | --- | --- | --- | --- | --- | --- | --- | --- | --- | --- | --- |
|  |  | **CON** | | **SYN1** | | **SYN2** | | **SYN3** |  |  |  |  |  |
| **Glucose (mg/dL)** | | | | | | | | | | | | |  |
| 0 day | | 95.00±1.05 | | 93.00±2.28 | | 91.70±1.71 | | 92.50±1.02 | 0.203 | <0.001 | 0.986 |  |  |
| 30 days | | 85.00±1.79 | | 82.40±2.18 | | 83.20±1.82 | | 84.00±1.53 |  |  |  |  |  |
| 60 days | | 79.20±1.80 | | 75.40±1.44 | | 76.70±1.63 | | 77.30±1.65 |  |  |  |  |  |
| Average | | 86.40±1.90 | | 83.60±2.21 | | 83.80±1.76 | | 84.60±1.69 |  |  |  |  |  |
| **Total protein (g/dL)** | | | | | | | | | | | | |  |
| 0 day | | 7.40±0.56 | | 6.73±0.47 | | 6.39±0.45 | | 6.72±0.49 | 0.424 | 0.996 | 0.977 |  |  |
| 30 days | | 7.72±0.51 | | 6.39±0.52 | | 6.70±0.85 | | 6.58±0.88 |  |  |  |  |  |
| 60 days | | 7.03±0.63 | | 6.81±0.76 | | 6.92±0.62 | | 6.55±0.56 |  |  |  |  |  |
| Average | | 7.38±0.31 | | 6.64±0.32 | | 6.67±0.36 | | 6.62±0.36 |  |  |  |  |  |
| **Albumin (g/dL)** | | | | | | | | | | | | |  |
| 0 day | 2.98±0.29 | | 3.02±0.44 | | 3.04±0.30 | | 2.41±0.31 | | 0.270 | 0.921 | 0.764 |  |  |
| 30 days | 2.66±0.30 | | 3.49±0.36 | | 2.64±0.43 | | 2.59±0.33 | |  |  |  |  |  |
| 60 days | 2.96±0.34 | | 2.86±0.45 | | 2.57±0.32 | | 2.67±0.33 | |  |  |  |  |  |
| Average | 2.87±0.17 | | 3.12±0.23 | | 2.75±0.20 | | 2.55±0.18 | |  |  |  |  |  |
| **Globulin (g/dL)** | | | | | | | | | | | | |  |
| 0 day | 4.41±0.64 | | 3.71±0.85 | | 3.34±0.47 | | 4.32±0.70 | | 0.482 | 0.978 | 0.807 |  |  |
| 30 days | 5.07±0.70 | | 2.90±0.82 | | 4.06±0.81 | | 3.99±0.77 | |  |  |  |  |  |
| 60 days | 4.07±0.85 | | 3.94±0.63 | | 4.34±0.79 | | 3.88±0.82 | |  |  |  |  |  |
| Average | 4.52±0.41 | | 3.52±0.43 | | 3.92±0.40 | | 4.06±0.42 | |  |  |  |  |  |
| **A: G** | | | | | | | | | | | | |  |
| 0 day | 0.75±0.15 | | 1.37±0.67 | | 1.05±0.22 | | 0.76±0.26 | | 0.868 | 0.810 | 0.799 |  |  |
| 30 days | 0.63±0.20 | | 0.99±0.18 | | 0.92±0.28 | | 0.81±0.21 | |  |  |  |  |  |
| 60 days | 1.01±0.35 | | 0.80±0.20 | | 0.80±0.24 | | 1.28±0.68 | |  |  |  |  |  |
| Average | 0.80±0.14 | | 1.05±0.23 | | 0.92±0.14 | | 0.95±0.25 | |  |  |  |  | |

**SM4:** Effect of synbiotics formulation on blood hematological indices of pre-ruminant buffalo calves after different time interval

| **Parameters** | **Dietary groups** | | | | **T** | **P** | **T*P** |  |
| --- | --- | --- | --- | --- | --- | --- | --- | --- |
|  | **CON** | **SYN1** | **SYN2** | **SYN3** |  |  |  |  |
| **Hemoglobin (g/dL)** | | | | | | | | |
| 0 day | 10.10±0.52 | 10.24±0.28 | 10.02±0.52 | 10.18±0.24 | 0.411 | 0.172 | 0.954 |  |
| 30 days | 10.30±0.37 | 10.40±0.49 | 10.45±0.51 | 10.92±0.51 |  |  |  |  |
| 60 days | 10.42±0.33 | 10.64±0.64 | 10.59±0.64 | 11.57±0.59 |  |  |  |  |
| Average | 10.27±0.22 | 10.43±0.27 | 10.35±0.31 | 10.89±0.29 |  |  |  |  |
| **PCV (%)** | | | | | | | | |
| 0 day | 31.60±1.50 | 33.80±1.69 | 30.17±0.87 | 32.50±1.12 | 0.704 | 0.030 | 0.670 |  |
| 30 days | 33.60±1.63 | 35.00±2.41 | 33.33±1.23 | 34.67±0.88 |  |  |  |  |
| 60 days | 34.00±1.14 | 34.20±1.39 | 36.17±1.68 | 34.67±1.82 |  |  |  |  |
| Average | 33.07±0.82 | 34.33±1.01 | 33.22±0.92 | 33.94±0.76 |  |  |  |  |
| **TEC (10^6^)** | | | | | | | | |
| 0 day | 7.90±0.33 | 8.20±0.31 | 8.15±0.38 | 8.34±0.38 | 0.263 | 0.691 | 0.999 |  |
| 30 days | 7.68±0.36 | 8.12±0.46 | 7.79±0.21 | 8.29±0.27 |  |  |  |  |
| 60 days | 7.88±0.28 | 8.42±0.62 | 7.97±0.27 | 8.44±0.42 |  |  |  |  |
| Average | 7.82±0.18 | 8.25±0.26 | 7.97±0.16 | 8.36±0.20 |  |  |  |  |

**SM5:** Effect of synbiotics formulation on blood hematological indices (DLC) of pre-ruminant buffalo calves after different time interval

| **Parameter** | **Dietary groups** | | | | |  | | | **P** | | | **T*P** |  |
| --- | --- | --- | --- | --- | --- | --- | --- | --- | --- | --- | --- | --- | --- |
|  | **CON** | | **SYN1** | **SYN2** | **SYN3** | | **T** | |  |  |  |  |  |
| **Neutrophils (%)** | | | | | | | | | | | | | |
| 0 day | 29.00±1.22 | 28.80±1.28 | | 29.30±0.84 | 29.70±1.28 | | | 0.970 | | 0.795 | | 0.967 |  |
| 30 days | 29.60±1.03 | 30.60±1.78 | | 29.00±0.58 | 29.80±1.30 | | |  | |  | |  |  |
| 60 days | 30.00±0.84 | 29.20±1.53 | | 29.20±1.17 | 29.20±1.05 | | |  | |  | |  |  |
| Average | 29.50±0.57 | 29.50±0.85 | | 29.20±0.49 | 29.60±0.66 | | |  | |  | |  |  |
| **Lymphocytes (%)** | | | | | | | | | | | | | |
| 0 day | 65.80±1.24 | 65.60±1.36 | | 65.7±1.15 | 65.30±1.20 | | | 0.996 | | 0.734 | | 0.975 |  |
| 30 days | 66.00±1.05 | 64.80±1.74 | | 65.2±0.95 | 64.80±1.17 | | |  | |  | |  |  |
| 60 days | 65.20±1.24 | 66.40±1.21 | | 65.7±0.99 | 66.20±0.95 | | |  | |  | |  |  |
| Average | 65.70±0.64 | 65.60±0.80 | | 65.5±0.56 | 65.40±0.62 | | |  | |  | |  |  |
| **Monocytes (%)** | | | | | | | | | | | | | |
| 0 day | 2.40±0.40 | 2.80±0.37 | | 2.50±0.34 | 2.50±0.43 | | | 0.770 | | 0.592 | | 0.839 |  |
| 30 days | 2.00±0.32 | 2.40±0.60 | | 2.83±0.31 | 2.67±0.33 | | |  | |  | |  |  |
| 60 days | 2.40±0.24 | 2.00±0.45 | | 2.50±0.34 | 2.17±0.54 | | |  | |  | |  |  |
| Average | 2.27±0.18 | 2.40±0.27 | | 2.61±0.18 | 2.44±0.25 | | |  | |  | |  |  |
| **Eosinophils (%)** | | | | | | | | | | | | | |
| 0 day | 2.80±0.37 | 2.60±0.24 | | 2.33±0.42 | 2.33±0.42 | | | 0.787 | | 0.315 | | 0.864 |  |
| 30 days | 2.20±0.49 | 1.80±0.37 | | 2.50±0.22 | 2.33±0.21 | | |  | |  | |  |  |
| 60 days | 2.20±0.37 | 2.00±0.32 | | 2.33±0.42 | 2.00±0.45 | | |  | |  | |  |  |
| Average | 2.40±0.24 | 2.13±0.19 | | 2.39±0.20 | 2.22±0.21 | | |  | |  | |  |  |
| **Basophils (%)** | | | | | | | | | | | | | |
| 0 day | 0.40±0.24 | 0.20±0.20 | | 0.33±0.21 | 0.17±0.17 | | | 0.923 | | 0.822 | | 0.886 |  |
| 30 days | 0.20±0.20 | 0.40±0.24 | | 0.50±0.22 | 0.33±0.21 | | |  | |  | |  |  |
| 60 days | 0.20±0.20 | 0.40±0.24 | | 0.33±0.21 | 0.50±0.22 | | |  | |  | |  |  |
| Average | 0.27±0.12 | 0.33±0.13 | | 0.39±0.12 | 0.33±0.11 | | |  | | |  |  |  |

**SM6:** Effect of synbiotics formulation on immunoglobulin and antioxidant enzyme activities in different groups of pre-ruminant buffalo calves (CON: Control), SYN1 (3g FOS+ *Lactobacillus plantarum* CRD7-100ml), SYN2 (6g FOS+ *Lactobacillus plantarum* CRD7-150ml) and SYN3 (9g FOS+ *Lactobacillus plantarum* CRD7-50ml)

| **Parameter** | **Dietary groups** | | | | |  | |  | | |  |
| --- | --- | --- | --- | --- | --- | --- | --- | --- | --- | --- | --- |
|  | **CON** | **SYN1** | **SYN2 SYN3** | | | | **T** | | **P** | **T*P** | |
| **IgG (mg/ml)** | | | | | | |  | |  |  | |
| 0 day | 8.77±0.87 | 9.80±1.04 | 9.42±0.74 | 8.71±0.60 | | 0.202 | | | 0.001 | 0.999 | |
| 30 days | 7.23±0.64 | 8.37±0.90 | 7.85±0.60 | 7.34±0.41 | |  | | |  |  | |
| 60 days | 6.79±0.42 | 7.72±0.75 | 7.76±0.55 | 7.08±0.41 | |  | | |  |  | |
| Average | 7.60±0.42 | 8.63±0.54 | 8.34±0.39 | 7.71±0.31 | |  | | |  |  | |
| **IgA (mg/ml)** | | | | | | |  | |  |  | |
| 0 day | 0.26±0.04 | 0.28±0.03 | 0.27±0.02 | 0.28±0.03 | 0.116 | | | | 0.742 | 0.906 | |
| 30 days | 0.24±0.02 | 0.25±0.01 | 0.29±0.01 | 0.30±0.02 |  | | | |  |  | |
| 60 days | 0.24±0.02 | 0.26±0.01 | 0.27±0.01 | 0.28±0.01 |  | | | |  |  | |
| Average | 0.25±0.02 | 0.27±0.01 | 0.28±0.01 | 0.29±0.01 |  | | | |  |  | |
| **SOD (U/mg Hb)** | | | | | | |  | |  |  | |
| 0 day | 47.50±1.76 | 48.90±1.82 | 49.00±1.56 | 48.70±1.39 | | | 0.027 | | 0.001 | 0.899 | |
| 30 days | 49.00±1.20 | 51.80±1.83 | 53.10±1.52 | 52.90±1.61 | | |  | |  |  | |
| 60 days | 49.30±1.41 | 54.00±1.38 | 54.90±1.29 | 54.00±1.83 | | |  | |  |  | |
| Average | 48.60^a^±0.42 | 51.60^ab^±1.06 | 52.30^b^±0.99 | 51.80^ab^±1.04 | | |  | |  |  | |
| **CAT (µmol of H_2_O_2_/min/g Hb)** | | | | | | |  | |  |  | |
| 0 day | 95.98±2.35 | 96.54±1.38 | 95.82±2.11 | 96.18±2.18 | | | 0.005 | | 0.013 | 0.643 | |
| 30 days | 96.56±2.26 | 100.06±1.62 | 102.83±1.41 | 99.55±1.99 | | |  | |  |  | |
| 60 days | 96.78^a^±2.82 | 100.26^ab^±1.34 | 105.05^b^±2.21 | 101.73^ab^±1.41 | | |  | |  |  | |
| Average | 96.44^a^±1.33 | 98.95^ab^±0.90 | 101.23^b^±1.42 | 99.16^ab^±1.16 | | |  | |  |  | |

Means bearing different superscripts a and b in the same row differ significantly (*P*<0.05)
